# Individuals who are ‘super recognisers’ show superior performance on independent measures of face perception, face memory, and face matching

**DOI:** 10.3758/s13423-024-02627-9

**Published:** 2025-09-10

**Authors:** Mirta Stantić, Zoë Pounder, Sarah Bate, Caroline Catmur, Geoffrey Bird

**Affiliations:** 1 Department of Experimental Psychology, University of Oxford, Oxford, UK; 2 Department of Psychology, Royall Holloway University of London, Egham, UK; 3 Department of Psychology, Bournemouth University, Poole, UK; 4 Department of Psychology, King’s College London, London, UK; 5 Centre for Research in Autism and Education, Institute of Education, University College London, London, UK

**Keywords:** Face perception, Face matching, Face memory, Super recogniser

## Abstract

Individuals who are superior at face recognition are described as ‘super recognisers’ (SRs). On standard face recognition tasks SRs outperform individuals who have typical face recognition ability. However, high accuracy on face recognition tasks may be driven by superior ability in one or more of several component processes including face perception, face matching, and face memory. The present study utilised the Oxford Face Matching Test (OFMT) and a novel analysis strategy to derive independent measures of face perception, face matching, and face memory. Thirty-two SRs and the same number of matched controls with typical face recognition ability undertook three face processing tasks: the OFMT, the Glasgow Face Matching Test, and the Cambridge Face Memory Test. At the group level, SRs were more accurate than controls across all tasks, and they reported greater face recognition ability. Of most importance, however, was the finding that SRs exhibited superior face perception, face matching, and face memory. Collectively, these results suggest that SRs have superior ability across multiple independent face-related processes.

## Introduction

Individuals who have an extraordinary ability to recognise faces are known as ‘super recognisers’ (SRs; e.g., Russell et al., 2009). As would be expected, many studies have confirmed that SRs outperform typical individuals on face recognition tasks. Such a finding does not explain *why* SRs have such good face recognition ability, however, and so it is interesting that several studies report superior performance by SRs, or at least a subset of SRs, on other face processing tasks (Belanova et al., 2018; Bobak et al., 2016a, b; Smith et al., 2021). They excel on tests of face memory and have been found in some studies to show superior performance on tests of face perception (designed to assess the ability to form an accurate representation of faces, often based on degraded input; Bobak et al., 2016a; Russell et al., 2009), and a subset of SRs have been found to perform at a superior level on face matching tasks (in which participants are required to determine whether two facial images are of the same person or of different people; Bate et al., 2018; Bobak et al., 2016a).

Recent work has attempted to explain the superior face recognition skills exhibited by SRs by examining their face processing strategies, For instance, SRs have been found to use facial information more effectively and consistently than typical perceivers (Nador et al., 2021; Tardif et al., 2019). This is achieved using feature-based processing strategies to more effectively process faces (Abudarham et al., 2021; see also Yovel et al., 2024) alongside a more widely distributed fixation pattern in their facial information sampling—a technique that may build a more holistic representation of the face (Dunn et al., 2022; see also Bobak et al., 2017). While these findings offer important information as to the correlates of superior face recognition skills, it has been more challenging to directly test hypotheses about reasons for SR ability with objective tests of face processing ability, given that such tests are not ‘process-pure’—that is, they do not allow performance on a single aspect of face processing to be tested independently of other processes. For example, face memory tasks, such as the Cambridge Face Memory Test (CFMT; Duchaine & Nakayama, 2006), require participants to identify a prelearned ‘target’ facial identity among distractors. While face memory is necessary to perform accurately on this task, face perception and face matching are also required. Face perception is required for the target face representations (which are stored in memory) to be accurate, and for accurate representations of stimuli to be formed so that they can be compared with the stored target representations. Face matching (the psychological decision-making process whereby one decides whether two facial images are of the same facial identity or different identities) is also required to determine whether a stimulus face matches the target facial identity stored in memory or not.

Similarly, in so-called face matching tasks (e.g., Glasgow Face Matching Test [GFMT]; Burton et al., 2010), participants are presented with two face images simultaneously and are required to judge whether the images are of the same person or different people. Obviously, face matching tasks require the psychological process of face matching (deciding whether the two images are of the same or different identities), but they also require face perception so that accurate representations of the two faces can be formed. Even face perception tasks, such as the Cambridge Face Perception Test (CFPT; Duchaine et al., 2007), are not process pure. The CFPT requires participants to order face stimuli in terms of their similarity to a target face, where stimuli are derived by morphing the target face with another identity in varying proportions. Thus, more perceptually similar images also contain more of the same identity versus a different identity, making it impossible to determine whether face perception, face matching, or a combination of the two, is driving performance. Thus far, therefore, research has lacked the means to obtain independent measures of face perception, face matching, and face memory, to determine whether SRs show superior performance in each of these processes.

A strategy to obtain independent estimates of these processes has recently been developed, which relies on the use of the Oxford Face Matching Test (OFMT; Stantić, Brewer, et al., 2022). Participants completing the OFMT are presented with two faces and asked to rate the perceptual similarity of the two faces before deciding whether the two face images are of the same identity or different identities. The similarity of the faces is assessed using three leading face recognition algorithms, and the mean rating is taken as an objective measure of the degree of similarity of the faces (avoiding the bias towards average, neurotypical performance obtained using ‘wisdom of the crowd’ approaches; see Jeckeln et al., 2023; Stantić, Brewer, et al., 2022). Participants’ similarity ratings can be compared with the objective measure as an index of their face perception ability independent of their ability to determine whether the face images are of the same person (i.e., independent of face matching). In addition, face matching performance can be assessed while statistically controlling for face perception ability, providing an independent measure of face matching, and both face perception and face matching scores can be regressed onto CFMT (memory) scores, such that any variance not explained by face perception and face matching can be used as an independent measure of face memory.

The OFMT has been used to assess differences in face processing abilities in a range of clinical and nonclinical groups (Stantić, Ichijo, et al., 2022b; Stantić, Pounder, et al., 2022c; Stantić et al., 2021, 2023), including individuals with autism (Stantić, Ichijo, et al., 2022b; Stantić et al., 2023) and developmental prosopagnosia (Stantić, Pounder, et al., 2022c). Individuals with autism have frequently been demonstrated to have difficulties with face recognition (see Wiegelt et al., 2012), and the use of the OFMT and the analysis technique described above revealed difficulties with both face memory and face perception in the presence of intact face matching. In contrast, individuals with developmental prosopagnosia (characterised by life-long difficulties in face recognition) were shown to have impaired face memory, face perception, and face matching.

Super recognition ability and developmental prosopagnosia are often described as being at opposite ends of the typical face recognition spectrum (Bobak et al., 2017) despite there being little evidence to support this idea. One reason for the lack of evidence is the many issues with existing tests used in this line of research (see Bobak et al., 2023, for a recent discussion). Another reason is that SRs may not be one homogeneous group. For example, with respect to SRs’ superior recognition abilities are domain-general (i.e., their superior recognition ability applies to all visual stimuli rather than being specific to faces) or domain-specific, evidence suggests some SRs show domain-specificity and others show enhancement in visual recognition across domains (Bobak et al., 2016a; see Hendel et al., 2019, for evidence of specificity in very good perceivers, but not SRs). Fundamentally, what is needed are studies probing exactly why individuals with developmental prosopagnosia have such poor face recognition abilities and whether it is those same processes which underlie the superior face recognition abilities seen in SRs. Given the work described above, which demonstrated impaired face memory, perception, and matching in individuals with developmental prosopagnosia, if SRs are truly at the opposite end of the typical face processing spectrum, one would expect superior face memory, perception, and matching in SRs. It is this possibility that the current work was designed to test.

In summary, the current study uses the OFMT and the CFMT to derive independent measures of face perception, face matching, and face memory in a group of SRs and a matched control group with typical face processing ability to identify the specific processes that are superior in SRs. Participants also completed the Glasgow Face Matching Test to provide convergent validity to the results obtained using the OFMT.

## Methods

### Participants

Thirty-two SRs were recruited to participate in the study (16 women, 16 men: mean age = 39.9 years, *SD* = 9.3—note a subset of these data have been reported previously in Stantić, Brewer, et al., 2022). Super recogniser participants were recruited from a preexisting database of SRs who self-reported a superior ability to recognise faces and met the criteria for super recognition as assessed by performance on three established measures, consistent with new frameworks that recommend using multiple difficult ecologically valid tests to identify SRs (Bate et al., 2018; Ramon, 2021). Participants scored above the established cut-offs, calculated as more than 1.96 standard deviations higher than the neurotypical population, on at least two out of three of the following measures: the Cambridge Face Memory Test Long Form (CFMT + ; all SR participants scored above 90, *M* = 95.50, *SD* = 2.96, range: 91–102; Russell et al., 2009), the Models Memory Test (MMT; 23 participants scored above 73, *M* = 76.51, *SD* = 5.37, range: 68–88; Bate et al., 2018), and the Pairs Matching Test (PMT; 18 participants scored above 40, *M* = 40.03, *SD* = 3.76, range: 30–45; Bate et al., 2018).

An age and gender-matched sample of 32 neurotypical participants were recruited via Prolific.sc. Six of these participants were excluded because they exhibited a pattern of performance suggesting possible prosopagnosia (poor performance; *n* = 3, *M* = 31.67, *SD* = 5.13) or that they were an SR (superior performance; *n* = 3, all scored 69) on the CFMT. These participants were replaced with six new neurotypical participants who were again recruited via Prolific (neurotypical control *n* = 32; 16 women, 16 men: mean age = 39.1 years, *SD* = 5.8). The SR and neurotypical controls did not differ significantly in terms of age, *t*(62) = 0.41,* p* = 0.68, or gender distribution, χ^2^(1) = 0.00, *p* = 1. No participants from either group were excluded for failing attention check trials on the OFMT. All participants reported having normal or corrected-to-normal vision. All experimental protocols were approved by the Central University Research Ethics Committee, University of Oxford. All methods used in this study were carried out in accordance with the tenets of the Declaration of Helsinki.

### Procedure

In a randomized order, participants undertook two standard tests, one of face memory (CFMT; Duchaine & Nakayama, 2006) and a standard face matching task (GFMT; Burton et al., 2010) as well as the newer OFMT (Stantić, Brewer, et al., 2022). For details of each of the tasks, see below. SRs did not complete the CFMT during the testing session as the CFMT + was completed during a diagnostic testing session, from which the CFMT scores were calculated for comparison with typical perceivers, and CFMT + scores were used for validation of SR status. All tasks were completed online using the platform Gorilla (www.gorilla.sc). Informed consent was obtained from all participants. Data acquisition took place in 2019 and 2020.

### Oxford Face Matching Test (OFMT)

The OFMT (Stantić, Brewer, et al., 2022) is a novel face matching task. It comprises 100 match (i.e., same) and 100 mismatch (i.e., different) face pairs, with a maximum face matching score of 200. Participants are shown a face pair for 1,600 ms and are subsequently required to make two responses: (1) to provide a perceptual similarity judgment for each pair of faces on a scale from 0 to 100 (from *very dissimilar* to *very similar*); (2) to decide whether the faces are of the same person (or not). The OFMT is deliberately constructed such that faces in match and mismatch trials contain overlapping similarity distributions. Thus, two images of the same person can be perceptually markedly different, while images of two different individuals can be perceptually very similar. This allows for perceptual similarity to be dissociated from the outcome of a face matching process. The task also included 12 attention check trials that were designed to be answered correctly, even by individuals with severe face processing impairments. Participants were excluded if they answered two or more of these trials incorrectly. The OFMT (see Stantić, Brewer, et al., 2022, for a visual depiction of trials) is available to researchers on Gorilla for noncommercial use upon request (see Gorilla Open Materials repository: https://gorilla.sc/openmaterials/134286).

### Glasgow Face Matching Test (GFMT)

The GFMT (Burton et al., 2010) is a well-known face matching task and comprises 20 match and 20 mismatch trials. During the task, participants are presented with face pairs and are asked to respond whether the two faces are the same or different. The maximum possible score is 40.

### Cambridge Face Memory Test (CFMT)

The CFMT (Duchaine & Nakayama, 2006) is a commonly used face memory task. Participants initially learn six faces and are tested in three stages of increasing difficulty on three-alternative forced-choice trials. These trials contain two novel images and one image of a previously learnt identity. In the first stage, 18 trials are shown with no changes to viewpoint or lighting. In the second stage, 30 trials are shown with changes to viewpoint and lighting, and the final 24 trials are shown with an addition of visual noise and changes to viewpoint and lighting. The maximum possible score is 72.

### Analysis strategy

The analysis approach used here has been used to investigate the independent contributions of face perception, matching, and memory in autism (Stantić et al., 2023) and developmental prosopagnosia (Stantić, Pounder, et al., 2022c). This approach requires the two groups (SR and neurotypical controls) to be compared on each of face perception, face matching, and face memory, independent of the other two processes. In brief, an independent measure of face *perception* is obtained by comparing participants’ ratings of similarity for face pairs in the OFMT with the similarity ratings of (those same face pairs) derived from the average of three leading facial recognition algorithms (see Stantić, Brewer, et al., 2022; Stantić, Pounder, et al., 2022c, for more detail). Three leading commercially available algorithms (AWS Rekognition, FaceSoft, Azure Face Recognition) were used to assess each face pair on the OFMT for similarity, and these indices were then averaged to provide a more stable measure of pair similarity. These similarity indices were normalized from 0–1 to 0–100 (*very dissimilar* to *very similar*) to allow for mathematical comparison between human-provided and algorithmically derived similarities. This mean algorithmic similarity value is then compared with each participant’s rating across trials to generate an average absolute deviation from algorithmically provided similarity for each participant. This value represents the average difference between a participant’s similarity score and the average similarity score provided by the algorithms (see Stantić, Brewer, et al., 2022; Stantić, Pounder, et al., 2022c; Stantić et al., 2023).

Any effect of group on face *matching* independent of face perception was investigated by regressing either OFMT accuracy scores or GFMT accuracy scores on average algorithmic deviations and group (SR vs control). This allows an effect of group on face matching to be detected while face perception is controlled for statistically (Stantić, Pounder, et al., 2022c; Stantić et al., 2023).

By the same logic, any effect of group on face *memory* that is independent of both face perception *and* face matching can be obtained by regressing CFMT accuracy scores on average algorithmic deviations from the OFMT, face matching test scores from either the OFMT or GFMT, and group (SR vs control). Thus, in the current study, we explore whether individuals with SR have superior face perception, face matching (independent of face perception), and face memory (independent of face perception and face matching) using a series of regression analyses. In regression analyses that predict face matching, group (SR vs control), predictors (face perception), and their interaction were used to predict test scores (OFMT test scores). Including the interaction term in regression models allows for the relationship between face perception (i.e., algorithmic deviation) and OFMT scores to vary across groups. The restricted range of CFMT scores in the SR population violated the assumption of homoscedasticity for linear regressions, thus, CFMT residuals (independent of face perception and face matching) were computed. These residuals were calculated by regressing face matching (OFMT or GFMT) and face perception (deviation scores) onto CFMT scores, and the residuals from this analysis were compared between groups (SR vs control). Data were analysed using SPSS Statistics Version 28. Between-group comparisons (SR vs control) of participant demographics and individual task performance were analysed with parametric tests or the nonparametric equivalent when normality assumptions were violated. All statistical analyses were performed with a significance level of *p* < 0.05, and all between-groups analyses are reported with one-tailed *p* values. All data are available online (https://osf.io/zaurg/), and no part of the study procedures or analysis was formally preregistered, although analyses and procedures are identical to previous papers using the same approach with neurotypical and autistic individuals (Stantić et al., 2023) and individuals with developmental prosopagnosia (Stantić, Pounder, et al., 2022c).

## Results

Descriptive statistics split by participant group are included in Table 1.

**Table 1:** Descriptive statistics and correlations between all tasks separated by group (super recognisers or control) with Bonferroni-corrected *p* values

		***M* (*SD*)**	**CFMT**	**GFMT**	**OFMT**
**CFMT**	**SR**	70.9 (1.2)	–	–	
	**Control**	56.0 (9.6)	–	–	
**GFMT**	**SR**	38.5 (2.8)	0.10 (0.13)	–	–
	**Control**	33.0 (5.3)	0.23 (0.30)	–	–
**OFMT: Matching**	**SR**	163.8 (8.0)	0.27 (0.30)	0.17 (0.22)	–
	**Control**	149.9 (13.3)	0.50** (0.56)	0.45* (0.58)	–
**Algorithmic deviations**	**SR**	20.04 (4.38)	− 0.06	− 0.31	− 0.32
	**Control**	22.56 (2.48)	− 0.30	− 0.14	− 0.41*

One asterisk (*) denotes significance at the *p* < 0.025 level and two asterisks (**) at the *p* < 0.005 level. All Spearman correlations were performed as one-tailed analyses. Shown in parentheses are correlations between measures corrected for reliability of each individual measure (by dividing the observed correlation by the square root of the product of internal reliabilities for the two tests). We used reliability estimates for the largest available sample: 0.89 for CFMT + (Bate et al., 2021), 0.67 for GFMT (Bobak et al., 2023) and our own reliability estimate for the OFMT at 0.90 from a sample of 989 participants from Stantić et al. (2021)

### Group comparisons: Standard test scores

#### OFMT: Matching performance

**Fig. 1:**
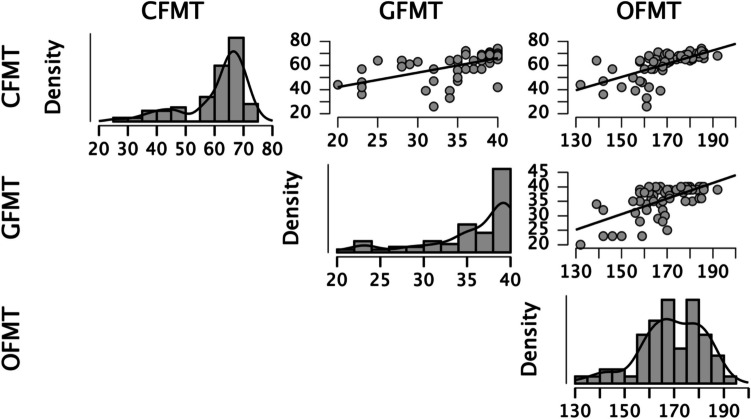
Relationships between key variables depicted for easier visualization. CFMT = Cambridge Face Memory Test; GFMT = Glasgow Face Matching Test; OFMT = Oxford Face Matching Test. OFMT scores are shown here (see Fig. 2, bottom right panel for algorithmic deviation scores). Each dot in the scatterplots indicates one participant

Eighteen SR participants (56%) performed one standard deviation above the control group’s mean score. Another 11 SRs (34%) had OFMT scores that were greater than the neurotypical mean score (but less than one standard deviation above the control mean), while an additional three SRs (9%) had an OFMT score that was either the same (*n* = 1) or below (*n* = 2) the neurotypical mean score (but less than one standard deviation below the control mean). An independent *t* test showed a significant group difference in OFMT matching accuracy, *t*(62) = 5.05, *p* < 0.001, *d* = 1.26, with SRs being more accurate (*M* = 82%, *SD* = 4%) than control participants (*M* = 75%, *SD* = 7%; see Fig. 1, upper left panel).

Signal detection theory (Green & Swets, 1966; see Bobak et al., 2023, for a discussion of its use in face matching tasks) was used to characterise performance on the matching component of the OFMT, providing a measure of sensitivity (*d′*) and bias (criterion). A Mann–Whitney test showed a significant group difference in *d′* (U = 183.50, *p* < 0.001, *r* = 0.55), with the SR group showing greater sensitivity (SR *d′* median: 1.99, range: 1.26–2.33; Control *d′* median: 1.56, range: 0.71–2.64) than the control group. A Mann–Whitney test revealed no group differences in criterion scores (*U* = 421.50, *p* = 0.11, *r* = 0.15) between the two participant groups.

#### OFMT: Algorithmic deviation

Seven SR participants (22%) performed two standard deviations below (indicating more accurate performance) the control mean standard deviation score, while 13 SR participants (41%) performed one standard deviation below the control mean. Another eight SRs (25%) had a deviation score that was below the neurotypical deviation mean (but less than one standard deviation). One SR (3%) scored one standard deviation above the control mean, and three other SRs (9%) scored two or more standard deviations above the control mean. A Mann–Whitney test showed a significant group difference in algorithmic deviation (*U* = 214, *p* < 0.001, *r* = 0.50), with SR participants having better deviation scores, or smaller deviation from algorithmic judgments (median = 19.08, range: 14.06–36.08), than the control participants (median = 22.42, range: 16.99–28.82).

### GFMT

Twenty-five SR participants (78%) performed one standard deviation above the Control group’s mean score. Another six SRs (19%) had GFMT scores that were greater than the neurotypical mean score (but less than one standard deviation above the control mean), and one SR (3%) had a GFMT score of more than one standard deviation below the control mean. A Mann–Whitney test revealed a significant group difference in GFMT accuracy (*U* = 121, *p* < 0.001, *r* = 0.66), with SR participants being more accurate (median = 39, range: 25–40) than the control participants (median = 35, range: 20–40; see Fig. 1, upper right panel).

### CFMT

**Fig. 2:**
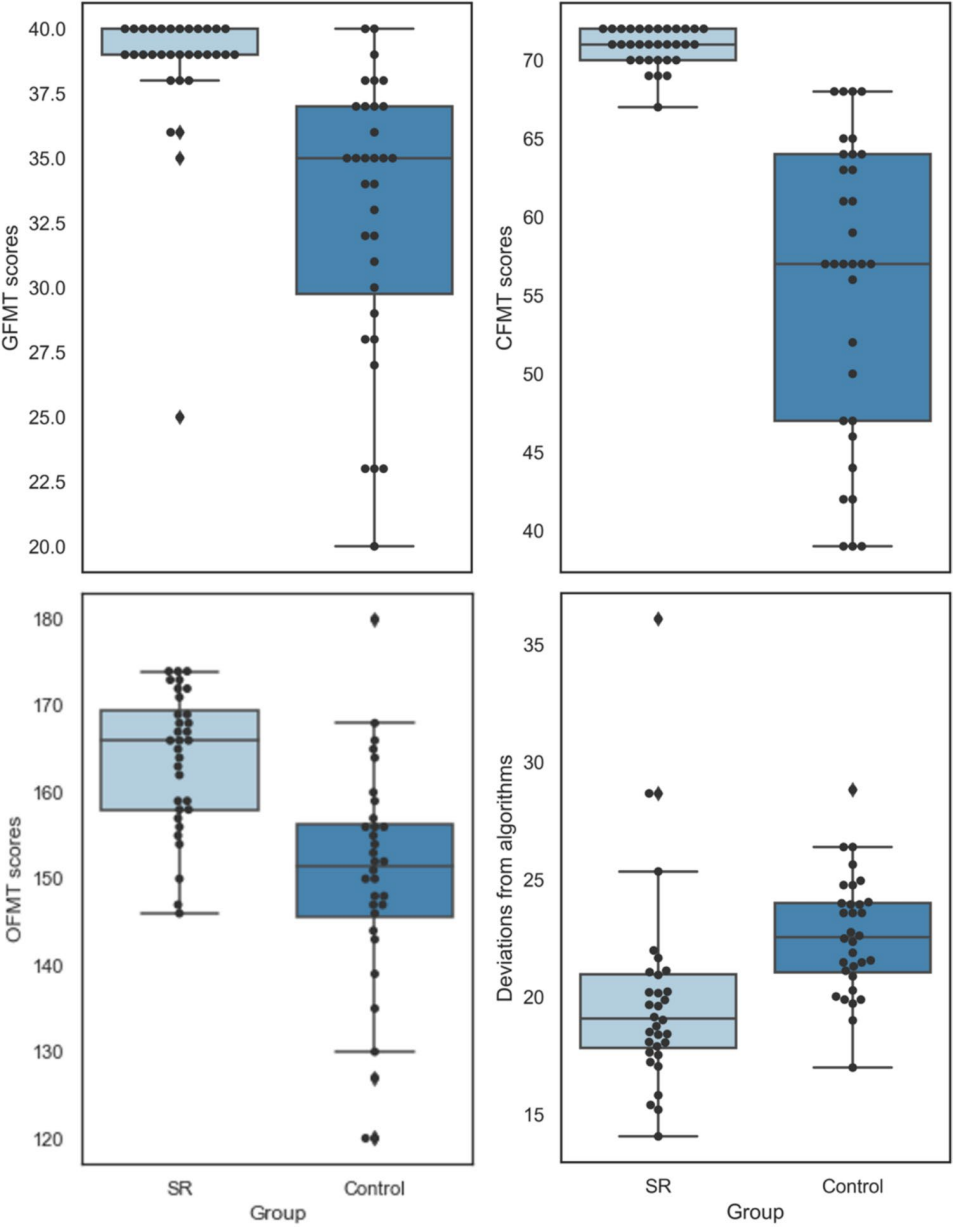
Raw scores for both groups shown by task. Top panel: GFMT scores and CFMT scores; bottom panel: OFMT scores and Algorithmic Deviations. Scores are expressed as percentages correct for OFMT, GFMT and CFMT, and deviations from algorithms are expressed as a raw score. Error bars represent confidence intervals set at 95%

**Fig. 3:**
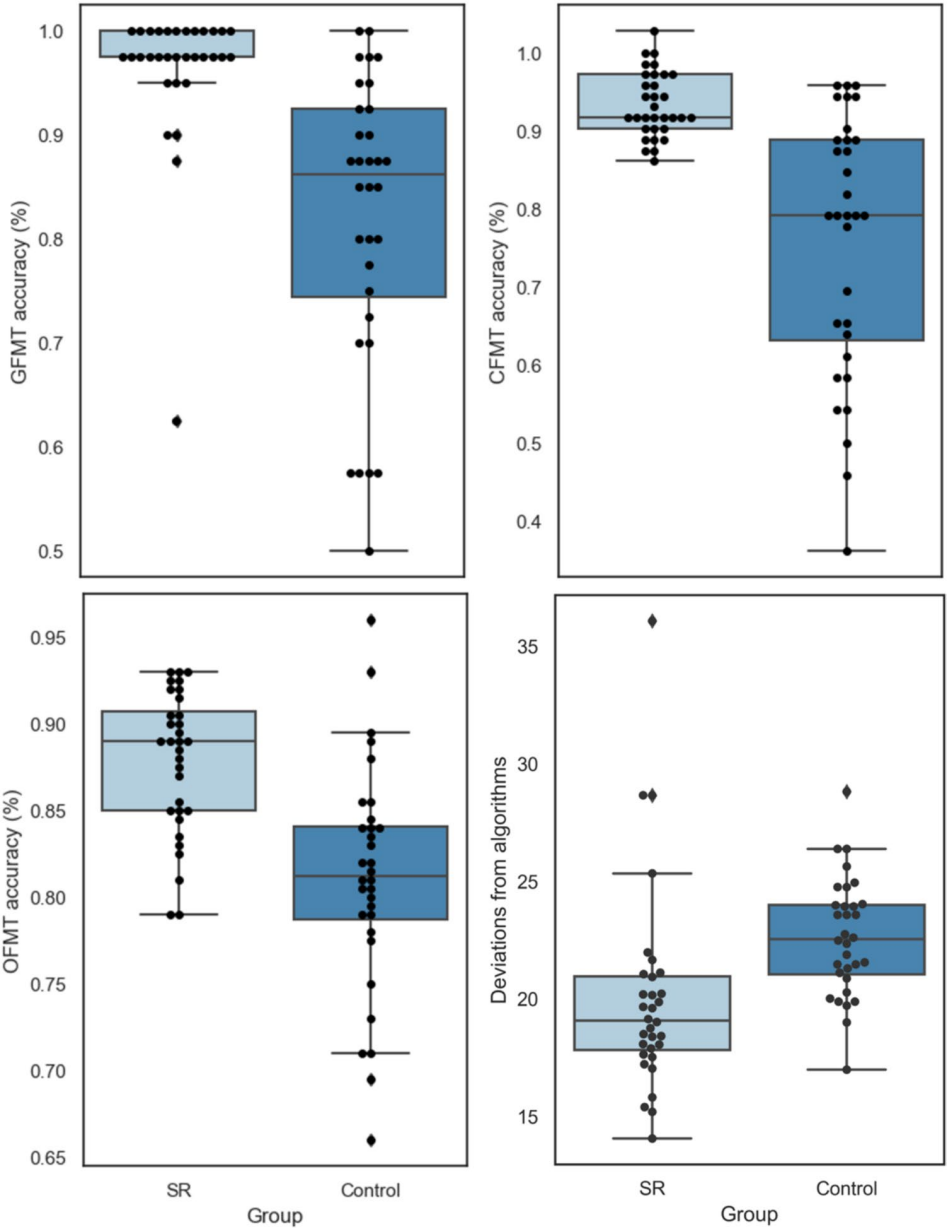
Raw scores for both groups shown by task. Top panel: GFMT scores and CFMT scores; bottom panel: OFMT scores and algorithmic deviations. Scores are expressed as percentages correct for OFMT, GFMT, and CFMT, and deviations from algorithms are expressed as a raw score. Error bars represent 95% confidence intervals

All 32 SR participants (100%) performed one standard deviation above the control group’s CFMT mean score. A Mann–Whitney test showed a significant group difference in CFMT accuracy (*U* = 4, *p* < 0.001, *r* = 0.86), with SR participants being more accurate (median = 71, range: 67–72) than the control participants (median = 57, range: 39–68; see Fig. 1, bottom right panel). This is, of course, expected given the selection criteria for inclusion in the SR group Figs. 2 and 3.

#### Face matching, controlling for face perception

Group (SR vs control), deviation scores, and their interaction were entered into regressions, the first predicting OFMT matching accuracy and the second, as a robustness check, predicting GFMT matching accuracy. For the OFMT analysis (adjusted *R*^2^ = 0.405), deviation scores were a significant predictor (β = − 0.42, *t* = − 3.38, *p* = 0.001) of OFMT matching accuracy, suggesting a relationship between face perception ability and performance on the OFMT. Group was also a significant predictor of OFMT matching accuracy (β = 0.40, *t* = 3.69, *p* < 0.001), indicating that face matching in the SR group was superior even when accounting for face perception ability. The interaction between group and deviation scores was not a significant predictor (β = 0.22, *t* = 1.89, *p* = 0.06).

This same analysis was repeated using GFMT instead of OFMT scores (adjusted *R*^2^ = 0.311). Group was again a significant predictor of GFMT matching accuracy (β = 0.52, *t* = 4.47, *p* < 0.001), suggesting that face matching was better in the SR group than in the control group after accounting for face perception ability. Deviation scores were not a significant predictor (β = − 0.10, *t* = − 0.73, *p* = 0.47), and the interaction between group and deviation was also not significant (β = 0.05, *t* = 0.39, *p* = 0.70).

#### Face memory, controlling for face perception, and face matching

The variance in CFMT scores in the SR group was smaller than the control group, violating the assumption of homoscedasticity for linear regression. Face matching (OFMT and GFMT separately) and deviation scores were therefore entered into a regression to predict CFMT scores (ignoring group status), and the unstandardised residuals from this analysis were compared between groups using a Mann–Whitney test.

For the OFMT, the Mann–Whitney test showed there was a significant difference in CFMT residuals (*U* = 177, *p* < 0.001, *r* = 0.56) with higher CFMT residuals in the SR group (median = 2.66, range: − 0.85–11.82) than the control group (median = − 3.35, range: − 22.65–17.04). Similarly, for CFMT residuals generated by regressing face matching GFMT scores and deviation scores on CFMT, a Mann–Whitney test showed there was a significant difference in CFMT residuals (*U* = 257, *p* < 0.001, *r* = 0.43), with higher CFMT residuals in the SR group (median = 2.68, range: − 0.94–17.07) than the control group (median = − 3.11, range: − 25.75–9.66).

Collectively, these results show that SRs exhibit greater CFMT performance compared with the control group, even after accounting for face perception (deviation scores) and face matching, suggesting that the SR group has better face memory than controls.

## Discussion

The primary aim of this paper was to compare SRs and individuals with typical face memory on face matching, face perception, and face memory. Results showed that, when assessed independently, SRs had superior ability in all three aspects of face processing. Furthermore, this pattern of data was replicated when matching scores for the GFMT were used instead of OFMT matching scores, indicating the robustness of these results. Group comparisons of performance on standard tests were aligned with this conclusion: The SR participants exhibited superior performance on all three face processing tests (OFMT, GFMT, and CFMT). SR participants also made similarity judgments of pairs of faces that were closer to those made by leading facial recognition algorithms than the matched control group.

When assessed at the group level, therefore, the idea that SRs are at the opposite end of the face recognition spectrum to developmental prosopagnosics is supported by these data, when compared with the performance of individuals with developmental prosopagnosia tested using the same approach (Stantić, Pounder, et al., 2022c). Both groups showed a consistent pattern of performance, such that they were either impaired or superior to average performers across face perception, matching and memory. Interestingly, this contrasts with individuals with autism who showed impaired face perception and memory, but intact face matching (Stantić et al., 2023).

Examination of the scores of individual SRs show, by definition, little variation in CFMT scores. Of more interest is the fact that significant variation is seen on face matching tests (particularly the OFMT, which avoids the problem frequently seen on the GFMT where ceiling effects are observed even among those with typical face processing ability, but especially with SRs; Dowsett & Burton, 2015; Dunn et al., 2020; Stantić, Brewer, et al., 2022; Verhallen et al., 2017). Future research should use additional difficult measures of matching, such as the Models Face Matching Test (Dowsett & Burton, 2015) or the Pairs Matching Test (Bate et al., 2018), to examine further the variance in face matching seen here with the OFMT among SRs. Additionally, it would be interesting for future research to examine whether measures that yield a wider range of face matching scores also show a relationship with algorithmically derived measures of face perception, as shown here for the OFMT. Even more variation is seen on the face perception measure (deviation of participants’ judgments from algorithmically derived similarity), indicating a wide range of differences between face similarity judged by algorithms and face similarity as judged by SRs.

The variation in face matching and face perception skill among individuals classed as SRs based primarily on their face recognition performance is of interest for both applied and theoretical reasons. In the applied domain, SR individuals are sometimes recruited by police forces and intelligence agencies. If employed on tasks requiring excellent face memory this may be perfectly appropriate, but if employed for other face processing tasks where performance is largely governed by face matching or face perception, some SRs may be no better than the average member of the public. Theoretically, this finding is of interest as it highlights the various routes by which one can achieve superior face recognition performance. Individuals can range from demonstrating exceptional face perception, matching and memory, to exceptional face memory only.

It is a limitation of this study, and indeed of the wider field, that appropriate control stimuli were not used to determine the degree to which superior performance is specific to faces, or common across stimulus categories (i.e., non-faces). It is possible, for example, that individuals who exhibit superior face memory may have excellent memory for all stimuli. In other words, these individuals may have hyperphantasia, an experience defined by the ability to represent previously experienced visual information with photograph-like precision and clarity (Zeman et al., 2020). Evidence consistent with this hypothesis exists at the opposite end of the imagery spectrum, where it is reported that some individuals who have an inability to represent visual information (known as aphantasia) also have prosopagnosia (Keogh et al., 2021; although see Monzel et al., 2023).

More broadly, the wide variation in face perception and face matching observed using the OFMT in the SR group strengthens the argument that the traditional single-measure approaches used to identify SRs in early research are not sufficient or at least do not identify a homogenous group. Even with administration of multiple ecologically valid tests along with the CFMT + , and only considering individuals as SRs if they perform at superior levels on multiple tests, we observe wide differences in perception and matching performance among SRs. It is worth considering whether research would benefit from studying superior performance across all of face perception, matching, and memory, perhaps by testing potential participants using an approach similar to that adopted here. Such as approach is supported by recent work showing that when a wide array of diagnostic tests is administered to a typical sample, correlations between different measures are low, and the consistency of participant performance across tests is poor (Bobak et al., 2023).

It should be noted however, that since the control group were not administered a CFMT + , only standard CFMT scores could be used for comparison of face memory between groups, restricting the variance of results for the SR group and therefore limiting scope for any individual-level analyses of scores on these tests. In addition, the newer version of the GFMT would have likely provided greater variance in matching scores than the original version used here; however, as this measure was not developed at the time of testing, it was not used.

Overall, this study derives independent measures of face perception, face matching, and face memory in a group of SRs and in a matched control group. Data indicate that, as a group, SRs have superior face perception, matching, and memory, while also revealing individual differences within the SR group in face perception and face matching.

## Data Availability

The datasets generated and/or analysed during the current study are available via the OSF repository (https://osf.io/zaurg/).

## Code availability

N/A.

## Declarations

### Open practices statement

All data are available online (https://osf.io/zaurg/). Neither study was preregistered.
